# Potential Antitonsillitis Metabolites From Endophytic Bacteria Associated With *Aporosa octandra* var. *malesiana*: A Metabolomic and Molecular Docking Study

**DOI:** 10.1155/ijm/7582229

**Published:** 2026-07-17

**Authors:** Zulfarina, Asih Rahayu Ajeng Agesti, Nursal, Syafrizal Ulum, Sipriyadi

**Affiliations:** ^1^ Department of Biology Education, Faculty of Teacher Training and Education, University of Riau, Pekanbaru, Indonesia, unri.ac.id; ^2^ Department of Biology, Faculty of Mathematics and Natural Science, University of Bengkulu, Bengkulu, Indonesia, unib.ac.id

**Keywords:** bioactive compounds, endophytic bacteria, native Sumatran plants, natural product

## Abstract

*Aporosa octandra* var. *malesiana* is a native Sumatran plant traditionally used in treating throat‐related conditions, indicating a rich reservoir of bioactive constituents. Endophytic microorganisms are known to produce metabolites similar to those found in medicinal plants. Therefore, this study explores the metabolomic fingerprint and antibacterial activity of an endophytic bacterium isolated from *Aporosa octandra* var. *malesiana* and examines its molecular interactions with key antitonsillitis targets through in silico approaches. In total, 44 endophytic bacterial isolates were obtained, of which 12 exhibited antagonistic activity against *Streptococcus pyogenes*. 16S rRNA gene–based molecular and phylogenetic analyses identified the isolate as *Streptomyces luteolus*. Metabolomic profiling using LC–MS/MS identified 4‐(dimethylamino)pyridine (4‐DMAP) and nicotinic acid as the two predominant metabolites. In silico docking analysis showed that these compounds interact with DNA gyrase and penicillin‐binding protein via hydrogen bonding and hydrophobic interactions, indicating their potential to inhibit essential bacterial processes. These results highlight *Streptomyces luteolus* associated with *Aporosa octandra* var. *malesiana* as a promising reservoir of antibacterial metabolites and support further studies to evaluate their potential against tonsillitis‐associated pathogens.

## 1. Introduction

*Aporosa octandra* var. *malesiana* is a wild plant native to Sumatra. This species is classified as a tree and grows predominantly in wet tropical biomes. *Aporosa octandra* var. *malesiana*, locally known as “Pelangas,” has been traditionally utilized for both nutritional and medicinal purposes. The leaves are traditionally consumed as a daily vegetable by local communities in Sumatra [1, 2]. Apart from its benefits as a vegetable, this plant is also reported to have medicinal properties for the treatment of tonsillitis and diabetes [3, 4]. Phytochemical analyses have revealed that *A. octandra* contains tannins, saponins, flavonoids, terpenoids, and steroids, which possess antioxidant and antiaging activities [5–8].

Based on its documented efficacy in treating throat inflammation and its richness in bioactive phytochemicals, *A. octandra* var. *malesiana* represents a promising candidate for the development of plant‐derived therapeutics. It is recognized that tonsillitis remains a significant global and national health concern, especially in regions with high population density and limited access to healthcare [9–11]. Tonsillitis is a common upper respiratory infection in both children and adults, primarily caused by *Streptococcus pyogenes* [12]. However, it may also result from other viral and bacterial pathogens such as rhinovirus, adenovirus, coronavirus, *Staphylococcus aureus*, *Haemophilus influenzae*, and *Corynebacterium diphtheriae* [13, 14].

The treatment of tonsillitis frequently encompasses several strategies, including the use of antibiotics, analgesics, and the surgical intervention known as tonsillectomy. Antibiotics have been widely used to treat tonsillitis, but increased antibiotic use is causing concern about antimicrobial resistance that leads to treatment failure and causes side effects in the application of antibiotic therapy [15–17]. In contrast to antibiotics, tonsillectomy is often another alternative for treating tonsils. However, tonsillectomy has the potential to cause postoperative complications. Thus, there is a compelling need for nonsurgical alternatives, including naturally derived compounds that may represent promising candidates against *S. pyogenes* and provide less invasive therapeutic options [18].

The therapeutic potential of *A. octandra* var. *malesiana* as a treatment for tonsillitis can be explored by investigating the bioactive capabilities of its endophytic bacterial community. Endophytic bacteria from medicinal plants have emerged as promising sources of selective antimicrobial metabolites. Exploring its endophytic bacteria represents a strategic way for developing herb‐based therapeutics without further exploiting the plant itself. Residing asymptomatically within plant tissues, endophytic bacteria possess remarkable biosynthetic capabilities that enable the production of structurally diverse secondary metabolites exhibiting a wide spectrum of biological activities, including antibacterial, antimicrobial, anti‐inflammatory, antimalarial, cytotoxic, antifungal, antimycobacterial, and antitumor effects [19–22]. Endophytes isolated from medicinal plants possess remarkable therapeutic potential [23–26]. Recent findings also indicate that many antibacterial metabolites produced by endophytic bacteria exhibit selective activity against human pathogens [27–29], highlighting their potential as sources of novel agents against *S. pyogenes*–associated infections.

Metabolomic and in silico approaches represent advanced and increasingly adopted strategies for elucidating the biochemical capacity of endophytic bacteria in drug discovery. These integrative methods enable rapid identification of bioactive metabolites, prediction of molecular targets, and evaluation of pharmacological properties, thereby accelerating the development of endophyte‐derived therapeutic candidates [30, 31]. However, information regarding the bioactive potential of endophytic bacteria associated with *A. octandra* var. *malesiana* remains limited, particularly for their possible application against tonsillitis‐associated pathogens.

Endophytic bacteria associated with the leaves of *A. octandra* var. *malesiana* were investigated for their antibacterial efficacy against *S. pyogenes*. To comprehensively elucidate the bioactive potential of the most potent isolate, an integrated experimental and computational framework was employed. Metabolomic analysis was utilized to profile and identify secondary metabolites produced by the endophyte, while molecular docking simulations were performed to explore the interaction patterns and binding affinities of these metabolites toward critical bacterial targets, namely, DNA gyrase and penicillin‐binding protein (PBP). Collectively, this study expands the current understanding of the antibacterial potential and metabolomic characteristics of endophytic bacteria associated with *A. octandra* var. *malesiana* and provides preliminary evidence supporting their possible relevance against tonsillitis‐associated pathogens.

## 2. Materials and Methods

### 2.1. Sample Collection and Endophytic Bacteria Isolation

Healthy, symptom‐free whole leaves of *A. octandra* var. *malesiana* were collected from Riau, Indonesia. Surface sterilization was performed following previously described procedures with minor modifications [32, 33]. The leaves were washed thoroughly and aseptically cut into fragments of approximately 30–40 mm in length. Leaf fragments were sequentially immersed in 70% ethanol for 2 min and 1% sodium hypochlorite (NaOCl) for an additional 2 min, followed by five rinses with sterile distilled water. The effectiveness of surface sterilization was verified by placing sterilized leaf segments on nutrient agar (NA) plates and incubating them for 72 h to confirm the absence of epiphytic microbial growth. Subsequently, the sterilized tissues were homogenized in sterile distilled water (1:10, *w*/*v*) using a sterile mortar and pestle, and 100 *μ*L of the homogenate was spread onto NA plates and incubated at room temperature for 72 h to allow the growth of endophytic bacteria.

### 2.2. Antibacterial Test of Endophytic Bacteria

Antibacterial activity was determined using the spot antagonism assay [34]. *Streptococcus pyogenes* (ATCC 19615) was cultured and adjusted to a 0.5 McFarland standard (approximately 1.5 × 10^8^ CFU/mL). A bacterial suspension was evenly spread onto Mueller–Hinton agar plates.Subsequently, 20 *μ*L of the endophytic bacterial culture was spotted onto the agar surface and incubated at 37°C for 24 h. Aquadest served as the negative control, whereas ampicillin (10 *μ*g/disc) was used as the positive control. All experiments were performed in triplicate, and inhibition zones were measured in millimeters, excluding the diameter of the bacterial colony.

### 2.3. Morphology and Biochemistry Identification of Potential Endophytic Bacteria

Macroscopic observation includes the shape, size, color, edge, and elevation of the colony [35]. Microscopic examination was performed through Gram staining using conventional staining protocols, and the Gram reaction, along with cellular morphology, was observed under an Olympus microscope. Biochemical characterization of the endophytic bacterial isolates was conducted using a series of standard assays, including catalase, oxidase, starch hydrolysis, casein hydrolysis, gelatin hydrolysis, hydrogen sulfide (H_2_S) production, motility, and nitrate reduction [36].

### 2.4. 16S Ribosomal RNA (rRNA) Gene Amplification by the PCR Technique

Genomic DNA was extracted from endophytic bacterial isolates using the Presto Mini gDNA Bacteria Kit (Geneaid) according to the protocol. Amplification of the 16S rRNA gene (~1300 bp) was performed using universal primers 8F (5 ^′^‐AGA GTT TGA TCC TGG CTC AG‐3 ^′^) and 1492R (5 ^′^‐GGT TAC CTT GTT ACG ACT T‐3 ^′^). The PCR products were visualized by agarose gel electrophoresis, and the resulting amplicons were subsequently submitted for sequencing to PT. Genetika Sains, Indonesia.

### 2.5. Extraction of Bacterial Metabolites

A 24‐h culture of the selected endophytic bacterium was inoculated at 1% (*v*/*v*) into 2 L of sterile nutrient broth (NB) and incubated for 72 h at 28°C–29°C with shaking at 120 rpm to promote metabolite production. The culture broth was then extracted twice with an equal volume of ethyl acetate (1:1, *v*/*v*) by agitation at 150 rpm for 1 h. The combined ethyl acetate fractions were concentrated under reduced pressure using a rotary evaporator at 50°C to obtain the crude extract for subsequent analyses.

### 2.6. LC–MS/MS Analysis

Bacterial extracts (10 mg) were dissolved in 5 mL of ethanol and filtered through a 0.2‐*μ*m PTFE membrane prior to analysis. LC–MS/MS profiling was conducted using an ultrahigh‐performance liquid chromatography (UHPLC) system (Thermo Fisher Scientific) coupled with a C18 UHPLC column (2.1 × 100 mm). The movement phase consisted of Solvent A (water containing 0.1% formic acid) and Solvent B (acetonitrile containing 0.1% formic acid), delivered at a flow rate of 0.3 mL/min. The gradient elution program was set as follows: 0–1 min, 5%; 1–20 min, 5%–95%; 20–23 min, 95%; and 23–25 min, re‐equilibration to 5%. Ionization was performed in polarity‐switching mode. The column temperature was maintained at 30°C, and a 5 *μ*L aliquot of the sample was injected for each analysis. Mass spectra were acquired over an *m*/*z* range of 100–1500. Metabolites were tentatively identified based on their precursor ions and MS/MS fragmentation patterns by comparison with spectral databases (MassBank and PubChem).

### 2.7. In Silico Study of Endophytic Bacteria

#### 2.7.1. Prediction of Compound Bioactivity

Bioactivity of each compound was predicted using PASS Online (https://way2drug.com/PassOnline/). Every bioactivity showed Pa (probability active) and Pi (probability inactive). Only antibacterial bioactivity would be considered for further analysis.

#### 2.7.2. ADMET (Absorption, Distribution, Metabolism, Excretion, and Toxicity) Analysis and Drug‐Likeness Analysis

ADMET analysis was used to predict the pharmacokinetic properties and toxicology of each compound. The pKCSM webserver (https://biosig.lab.uq.edu.au/pkcsm/) was used for ADME analysis, and the ProTox 3.0 webserver (https://tox.charite.de/protox3/) was used for toxicology. Drug‐likeness analysis was performed to evaluate the physiochemical similarity of the compounds to recent drugs. Drug‐likeness analysis was performed using Lipinski′s rule of five with the SwissADME webserver (https://www.swissadme.ch/). Lipinski′s rule of five is a common analysis used to determine whether drug candidates are orally effective based on their physiochemical properties. The criteria are H − bond ≤ 5, acceptor H − bond ≤ 10, molecular weight ≤ 500 g/mol, and partition coefficient (*C* log *P*) ≤ 5 [37].

### 2.8. Molecular Docking Studies

Three‐dimensional structures of the compounds and positive control (ciprofloxacin and ampicillin) of each protein are collected from the PubChem database. Three‐dimensional structures of DNA gyrase (Protein Data Bank [PDB] ID: 1KZN) and PBP (PDB ID: 3HUN) were collected from the PDB database. Protein was prepared by removing the water molecule and unwanted ligand using Discovery Studio Visualizer 2019. Specific molecular docking is conducted with PyRx 0.8 using the AutoDock Vina Program. The target residues used to define the docking region for each protein were identified from the literature, and the specific grid boxes are provided in File S1. The result of molecular docking is the binding affinity and conformation of ligands and proteins. Every conformation of ligands and each protein was visualized using Discovery Studio Visualizer 2019.

## 3. Results

### 3.1. Isolation of Endophytic Bacteria

**Figure 1:**
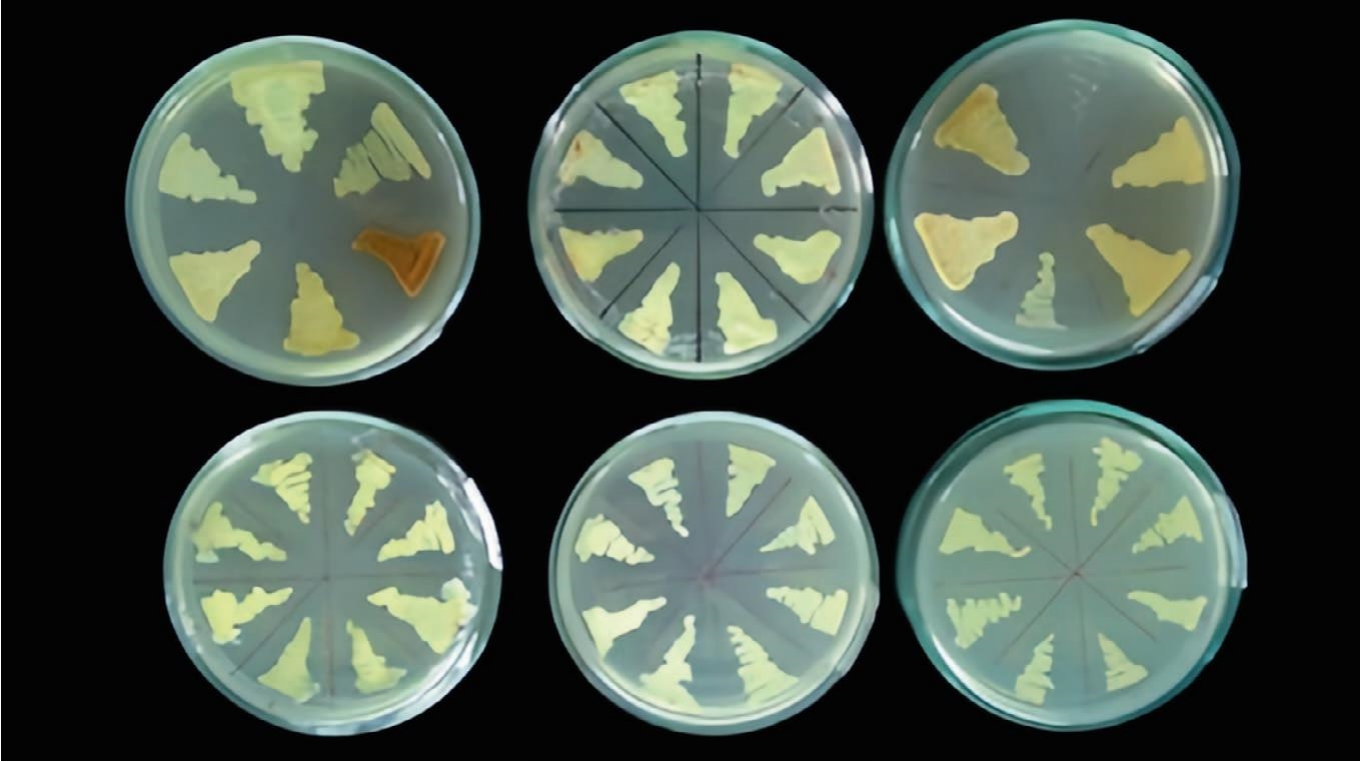
Endophytic bacteria isolated from surface‐sterilized leaves of *Aporosa octandra* var. *malesiana.*

Isolation of endophytic bacteria from the surface‐sterilized leaf tissues of *A. octandra* var. *malesiana* yielded 44 bacterial colonies with an incubation time of 72 h on NA medium (Figure 1). The absence of microbial growth in the imprint and final rinse controls confirmed the effectiveness of the surface sterilization procedure, indicating that the recovered colonies originated from internal plant tissues. The high number of colonies reflects the diversity and potential of the plant microbiome.

### 3.2. Antibacterial Activities of Endophytic Bacteria

**Figure 2:**
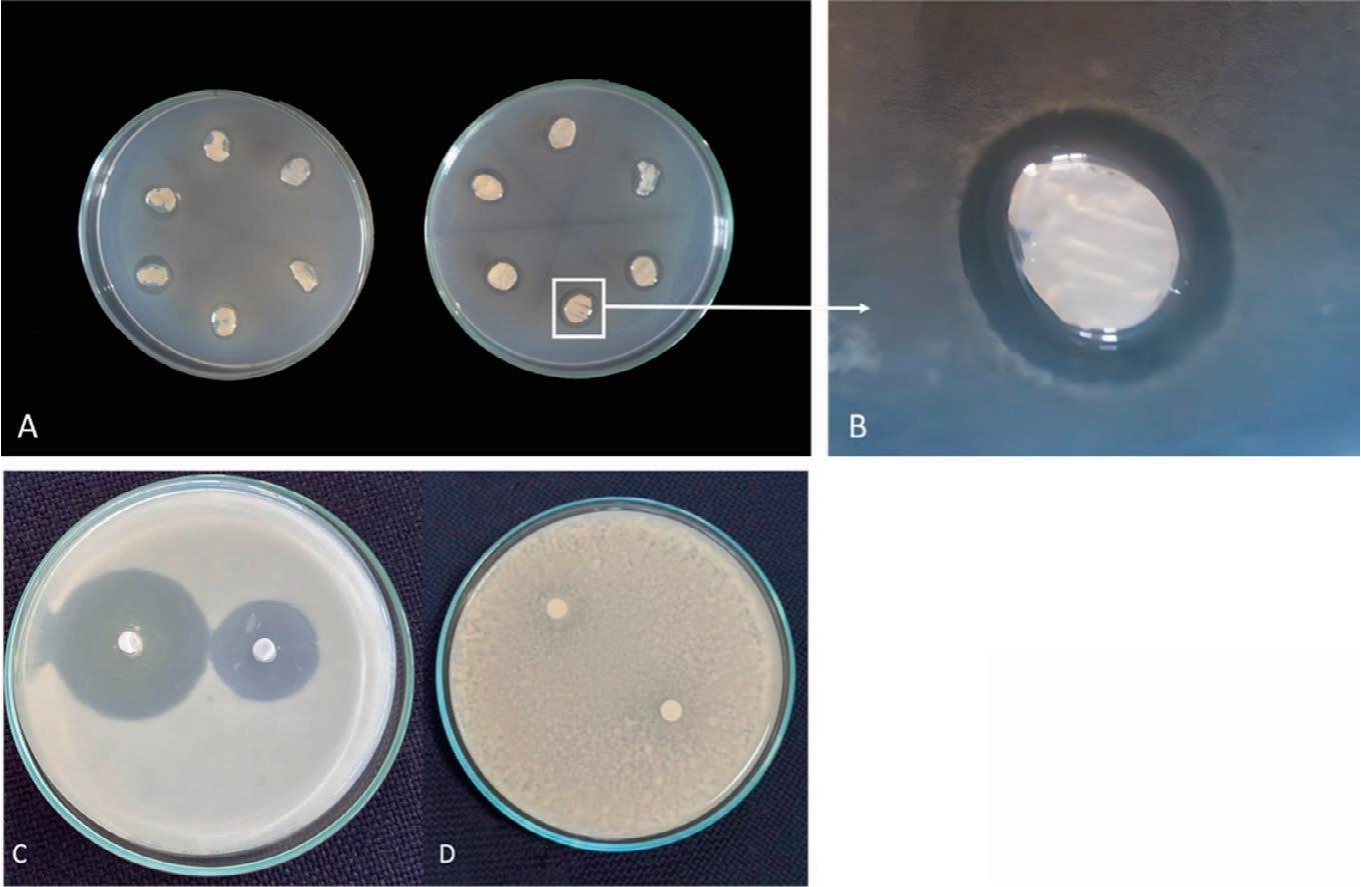
(A) Twelve endophytic bacterial isolates showing inhibitory activity against *Streptococcus pyogenes*. (B) The potential endophytic bacteria AO4. (C) Positive control (ampicillin) and (D) negative control (aquadest).

Antibacterial activity was evaluated for 44 endophytic bacterial isolates obtained from *A. octandra* var. *malesiana*. Twelve isolates exhibited measurable inhibitory activity against *S. pyogenes* (Figure 2). Inhibition zone diameters ranged from 3.3 ± 1.4 to 11.5 ± 2.4 mm, with Isolate AO4 displaying the strongest antagonistic effect (11.5 ± 2.4 mm), followed by AO12, AO5, and AO11 (Table 1 and Figure 2). Several isolates, including AO17, AO20, and AO32, showed relatively weak inhibition, with zones below 5 mm. Based on its superior antibacterial activity, Isolate AO4 was selected as the most promising candidate for further morphological, biochemical, molecular, and metabolomic characterization.

**Table 1:** The diameter of the inhibition zone of endophytic bacteria from *Aporosa octandra* var. *malesiana* against *Streptococcus pyogenes.*

No.	Isolates	Inhibition zone diameter (mm)
		*Streptococcus pyogenes*
1	AO2	5.2 ± 1.4
2	AO4	11.5 ± 2.4
3	AO5	9.4 ± 1.9
4	AO8	4.5 ± 0.9
5	AO11	8.9 ± 1.1
6	AO12	9.6 ± 0.7
7	AO17	3.7 ± 0.9
8	AO20	4.2 ± 0.7
9	AO22	4.9 ± 0.1
10	A032	3.3 ± 1.4
11	AO35	7.9 ± 0.7
12	AO40	6.1 ± 1.9
13	Positive control (ampicillin)	23 ± 0.1
14	Negative control (aquadest)	0 ± 0

### 3.3. Characterization of Potential Endophytic Bacteria

**Figure 3:**
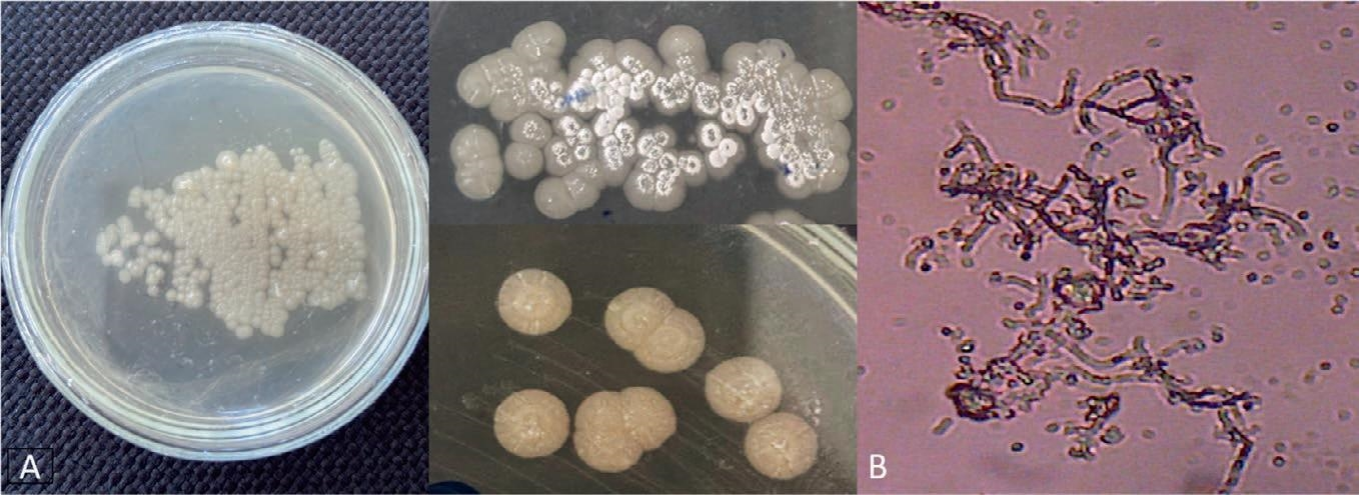
(A) Macroscopic colony characteristics of potential endophytic bacterial AO4 isolated from *Aporosa octandra* var. *malesiana.* (B) Gram staining of the colony shows Gram‐positive.

The most potent endophytic bacterial isolate (AO4) was further characterized based on its macroscopic, microscopic, and biochemical characteristics. The morphological and biochemical profile of the selected endophytic bacterium is summarized in Table 2. The isolate formed yellowish‐brown, chalky, circular colonies with entire margins and a convex elevation. Microscopic observation revealed a Gram‐positive, filamentous morphology with rectiflexible spore chains, consistent with actinomycete characteristics (Figure 3). Biochemical assays demonstrated strong enzymatic activity, with positive reactions for catalase, oxidase, starch, casein, and gelatin hydrolysis, alongside nitrate reduction. The isolate was nonmotile and negative for H_2_S production.

**Table 2:** Morphology and biochemistry characteristic of potential endophytic bacteria AO4 isolated from *Aporosa octandra* var. *malesiana.*

Category	Characters
Colony morphology	
Colony color	Yellowish brown
Texture	Chalky
Colony form	Circular
Margin	Entire
Elevation	Convex
Aerial mycelium	Cream
Substrate mycelium	Yellowish brown
Growth rate	72 h
Gram reaction	Gram‐positive
Cell shape	Filamentous
Spore formation	Rectiflexibles
Biochemical characteristics	
Catalase	Positive
Oxidase	Positive
Starch hydrolysis	Positive
Casein hydrolysis	Positive
Gelatin hydrolysis	Positive
H_2_S production	Negative
Motility	Nonmotile
Nitrate reduction	Positive

### 3.4. Identification of Potential Endophytic Bacteria

The 16S rRNA gene represents a highly conserved molecular marker widely employed for accurate bacterial phylogenetic classification. In this study, molecular characterization was conducted to elucidate the phylogenetic identity of an endophytic bacterial isolate associated with *A. octandra* var. *malesiana*. Genomic DNA from the isolate was amplified and sequenced targeting the 16S rRNA gene, and the resulting sequence was subjected to BLASTN analysis against curated sequences in the NCBI nucleotide database to determine its closest phylogenetic affiliations and taxonomic assignment.

Sequencing of the 16S rRNA gene from an isolate of endophytic bacteria produced a high‐quality sequence of approximately ~1300 bp. BLASTN analysis revealed that the isolate exhibited 100% query coverage and aligned most closely with *Streptomyces luteolus* strain B‐S‐A12, showing 100% sequence identity. Additional closely related taxa included *Streptomyces deserti* (99.67%), *S. monticola* (99.33%), and *S. limosus* (99.00%) (Table 3).

**Table 3:** NCBI BLAST analysis of 16S rRNA gene sequences showing the closest related species to endophytic bacterial Isolate AO4 from *Aporosa octandra* var. *malesiana.*

Isolate name	Description	Max score	Total score	Query cover	*E* value	Per. identity	Accession number
Kode AO4	*Streptomyces luteolus* strain B‐S‐A12	1105	1105	100%	0.0	100.00%	NR_199549.1
	*Streptomyces deserti* strain C63	1096	1096	100%	0.0	99.67%	NR_116355.1
	*Streptomyces monticola* strain NEAU‐GS4	1083	1083	100%	0.0	99.33%	NR_164930.1
	*Streptomyces limosus* strain NBRC 12790	1072	1072	100%	0.0	99.00%	NR_112279.1

**Figure 4:**
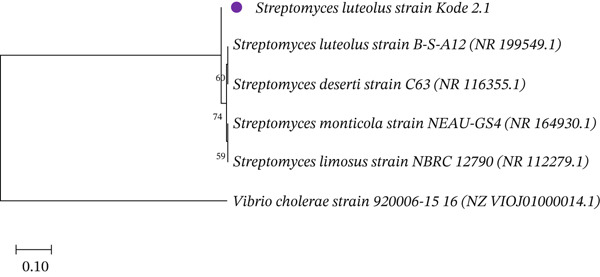
Phylogenetic tree based on 16S rRNA shows Isolate AO4 identified as *Streptomyces luteolus* strain Code 2.1.

Phylogenetic analysis of the 16S rRNA sequence was conducted in MEGA using ClustalW for alignment. The resulting topology showed that the isolate formed a strongly supported distinct monophyletic clade with *S. luteolus* strain B‐S‐A12, consistent with the BLASTN identification (Figure 4). The isolate also grouped closely with *S. deserti*, *S. monticola*, and *S. limosus*, separated by short branch lengths indicative of minor nucleotide divergence among these taxa. These results confirm the endophytic bacteria from *A. octandra* var. *malesiana* within the *S. luteolus* lineage. The 16S rRNA gene sequence of *S. luteolus* strain Kode 2.1 has been deposited in GenBank under accession number PZ303396.1.

### 3.5. Metabolomics Profiling by LC–MS/MS

**Figure 5:**
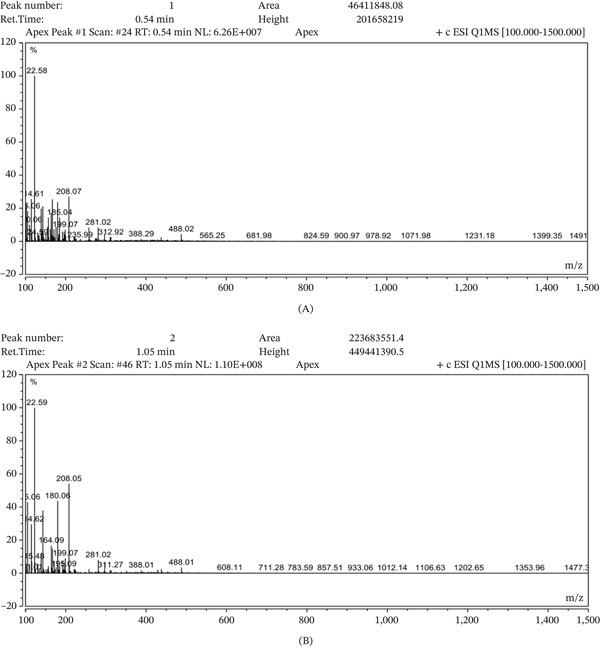
LC–MS chromatograms and mass spectra of the endophytic bacterial extract. (A) Peak 1 at a retention time of 0.54 min. (B) Peak 2 at a retention time of 1.05 min. Spectra were recorded in positive ESI mode (*m*/*z* 100–1500), showing distinct ion profiles for each detected peak.

LC–MS/MS analysis of the endophytic bacterial isolate revealed two major chromatographic peaks eluting at 0.538 and 1.053 min (Figure 5). Both peaks exhibited nearly identical mass spectral characteristics at *m*/*z* 122.581 (Peak 1) and *m*/*z* 122.593 (Peak 2). The similarity in retention and ionization indicates that the peaks correspond to closely related molecular constituents. The first compound is consistent with a monoisotopic mass of 122.0844 Da (molecular formula C_7_H_10_N_2_), which aligns with 4‐(dimethylamino)pyridine (4‐DMAP). The second identified compound corresponded to a monoisotopic mass of 123.0320 Da (molecular formula C_6_H_5_NO_2_), matching the spectral properties of nicotinic acid. Although several additional peaks were detected in the LC–MS/MS spectra, only metabolites with high‐confidence spectral matches were annotated and discussed in this study. These unannotated peaks may represent additional metabolites that contribute to the observed antibacterial activity and warrant further investigation.

### 3.6. In Silico of Endophytic Bacteria

**Figure 6:**
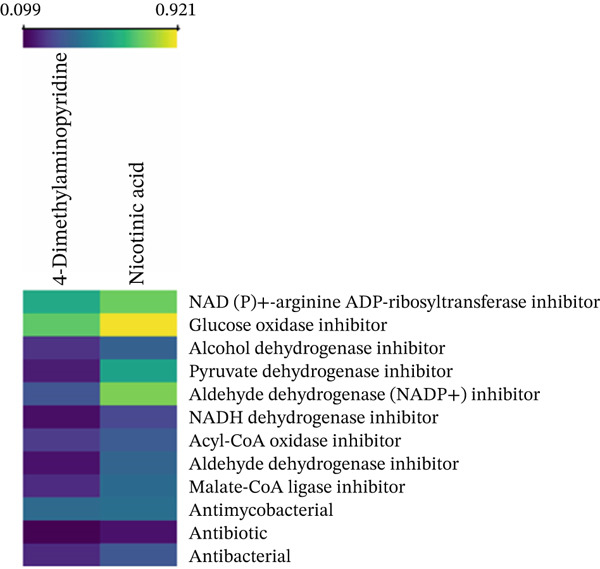
Bioactivity prediction of 4‐(dimethylamino)pyridine and nicotinic acid.

Bioactivity prediction was focused on antibacterial activity, including NAD(P)+arginine ADP‐ribosyltransferase inhibitor, glucose oxidase inhibitor, alcohol dehydrogenase inhibitor, pyruvate dehydrogenase inhibitor, aldehyde dehydrogenase (NADP+) inhibitor, NADH dehydrogenase inhibitor, acyl‐CoA oxidase inhibitor, aldehyde dehydrogenase inhibitor, malate‐CoA ligase inhibitor, antimycobacterial, antibiotic, and antibacterial. Pa value ranged from 0.099 to 0.921, with a higher Pa value representing a higher probability of bioactivity (Figure 6).

ADMET analysis is used to analyze the pharmacokinetic properties of compounds. Both 4‐DMAP and nicotinic acid exhibit high permeability compared to all three control compounds (Table 4). They are categorized with high Caco‐2 permeability, whereas all controls (ciprofloxacin and ampicillin) fall into the low/medium permeability class. Regarding solubility, 4‐DMAP stands out as the only compound classified as very soluble. Both test compounds also achieve high intestinal absorption (≥ 94%), comparable to ciprofloxacin.

**Table 4:** Pharmacokinetic analysis (ADMET) of tested compounds and control ligands.

Parameters of ADMET	4‐(Dimethylamino)pyridine	Nicotinic acid	Ciprofloxacin	Ampicillin
Absoprtion				
Water solubility (log mol/L)	−0.573	−2.134	−2.897	−2.396
Caco‐2 permeability	1.617	1.17	0.492	0.395
Intestinal absorption (%)	100	94.099	96.466	43.034
P‐glycoprotein substrate	Yes	No	Yes	No
P‐Glycoprotein I inhibitor	No	No	No	No
P‐Glycoprotein II inhibitor	No	No	No	No
Distribution				
VDss (log L/kg)	−0.08	−1.015	−0.17	−1.23
Fraction unbound	0.634	0.776	0.648	0.752
BBB permeability (log BB)	0.058	−0.323	−0.587	−0.767
CNS permeability (log PS)	−2.818	−2.869	−2.999	−3.166
Metabolism				
CYP2D6 substrate	No	No	No	No
CYP3A4 substrate	No	No	No	No
CYP1A2 inhibitor	No	No	No	No
CYP2C19 inhibitor	No	No	No	No
CYP2C9 inhibitor	No	No	No	No
CYP2D6 inhibitor	No	No	No	No
CYP3A4 inhibitor	No	No	No	No
Excretion				
Total clearance (log mL/min/kg)	0.567	0.652	0.633	0.337
Renal OCT2 substrate	No	No	No	No
Toxicity				
AMES toxicity	No	No	No	No
MRTD (mg/kg/day)	0.948	0.907	0.924	0.952
ORAT (mol/kg)	2.273	2.24	2.891	1.637
Hepatotoxicity	No	No	Yes	Yes
Carcinogenetic	0.85(1)	0.95(1)	0.57(1)	0.83(1)
LD_50_ (mg/kg)	250	3720	2000	5000
LD_50_ class	3	5	4	5

The test compounds and control compounds show diverse distribution characteristics. Nicotinic acid exhibits low distribution (VDss), aligning with ciprofloxacin and ampicillin, while 4‐DMAP has medium distribution. In terms of central nervous system (CNS) penetration, both test compounds are in the intermediate range. Both tested compounds are predicted to have moderate BBB penetration.

4‐DMAP and nicotinic acid possess a clean metabolic profile, as they are not predicted to be substrates or inhibitors of any of the major cytochrome P450 (CYP) enzymes (CYP1A2, CYP2C9, CYP2C19, CYP2D6, and CYP3A4). For excretion, none of the test or control compounds are predicted to be substrates for the renal OCT2 transporter. There is no compound which detected to have mutagenic activity. 4‐DMAP has a maximum dosage of 0.948 mg/kg/day, while the maximum dosage of nicotinic acid is 0.907 mg/kg/day. Each compound did not show toxicity to the hepar.

Drug‐likeness analysis is used to determine the similarity of potential compounds to current drugs. The similarity represented the effectiveness of compound absorption in the body. Thus, this analysis is crucial for screening potential compounds as drug candidates. Lipinski′s rule of five is a common analysis for determining drug candidates that work effectively orally. 4‐DMAP and nicotinic acid fulfilled the optimum criteria of Lipinski′s rule of five, with no violation of each criterion (Table 5).

**Table 5:** Drug likeness of Lipinski′s rule analysis.

Compounds	H‐bond donor	H‐bond acceptor	Molecular weight (g/mol)	*C* log *P*	Lipinski′s rule of five
Nicotinic acid	1	3	123.11	−1.13	Yes
4‐(Dimethylamino)pyridine	0	2	122.17	0.83	Yes

**Figure 7:**
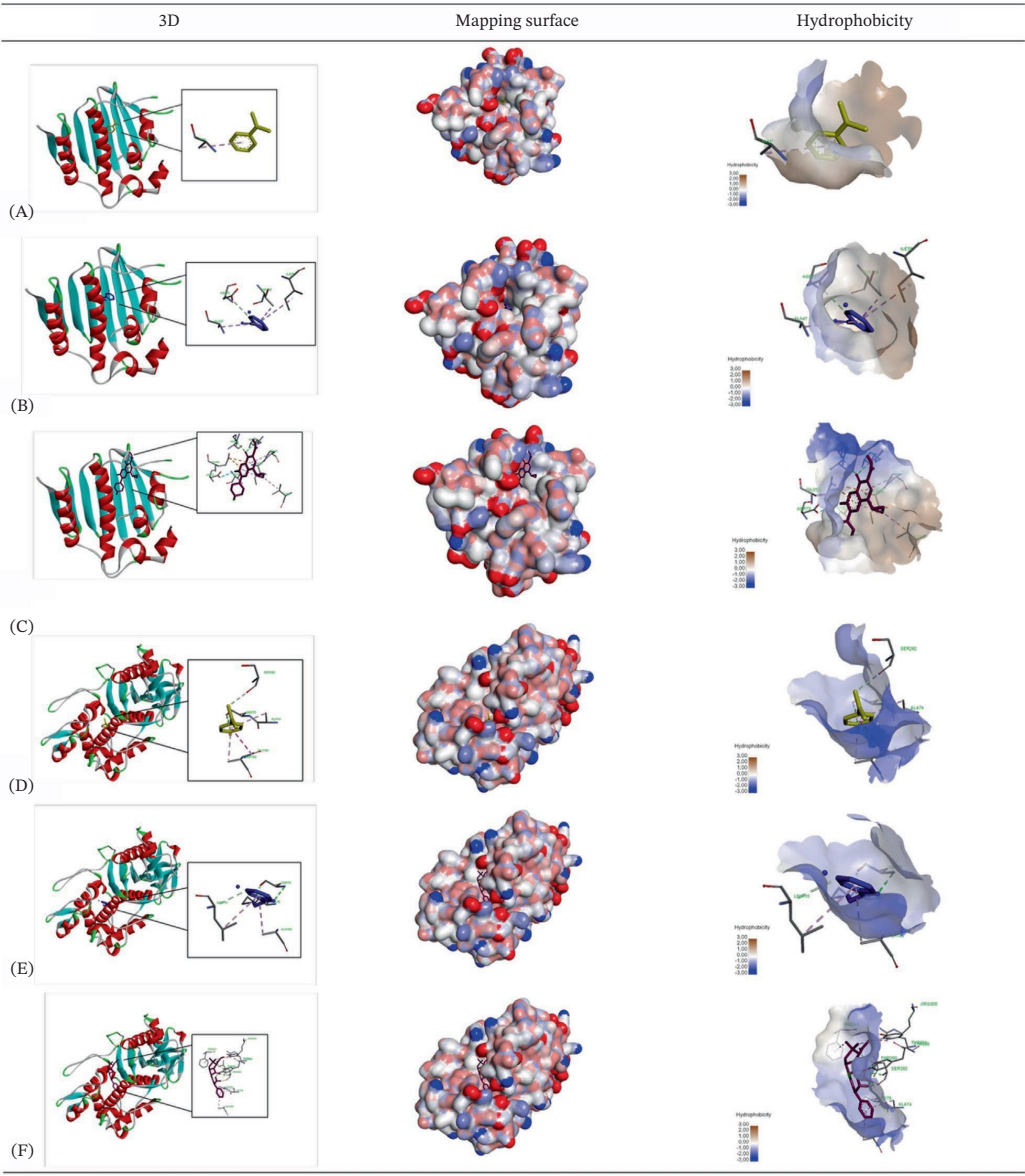
Interaction of test compounds and control ligands with the protein target. (A) The interaction of 4‐(dimethylamino)pyridine with DNA gyrase. (B) The interaction of nicotinic acid with DNA gyrase. (C) The interaction of ciprofloxacin with DNA gyrase. (D) The interaction of 4‐(dimethylamino)pyridine with penicillin‐binding protein. (E) The interaction of nicotinic acid with penicillin‐binding protein. (F) The interaction of ampicillin with penicillin‐binding protein.

Binding affinity represents the strength of protein–ligand interaction. Low binding affinity implies a strong interaction between protein and ligand. Based on Table 6, nicotinic acid has a higher binding affinity to DNA gyrase (−5.2 kcal/mol), while 4‐DMAP is −4.2 kcal/mol. However, ciprofloxacin has the lowest binding affinity to DNA gyrase (−7.6 kcal/mol). Moreover, nicotinic acid has a higher binding affinity to PBP (−5.3 kcal/mol) than 4‐DMAP. The binding of ligand to protein target, protein surface, and hydrophobicity is shown in Figure 7. For the protein target DNA gyrase, 4‐DMAP interacted only with Ala47 through a Pi‐alkyl hydrophobic contact, whereas nicotinic acid formed additional interactions with Asp73, Thr165, Ala47, and Ile78. PBP showed that 4‐DMAP formed hydrogen bonds with Ser75 and several hydrophobic interactions with Ser262, Gly181, Ala182, and Ala74, while nicotinic acid interacted with Ser75, Leu115, and Ala182.

**Table 6:** Binding affinity of each compound with protein target (DNA gyrase and penicillin‐binding protein).

Compounds	Binding affinity (kcal/mol)	
	DNA gyrase (1KZN)	Penicillin‐binding protein (3HUN)
4‐(Dimethylamino)pyridine	−4.2	−4.3
Nicotinic acid	−5.2	−5.3
Ciprofloxacin	−7.6	—
Ampicillin	—	−7.5

## 4. Discussion

Endophytic bacteria colonize plant tissues asymptomatically, utilizing plant‐derived resources while maintaining a mutualistic association. A total of 44 endophytic bacterial isolates were successfully obtained from the leaves of *A. octandra* var. *malesiana*. The isolation of this number of distinct strains highlights the substantial capacity of the host plant to support diverse endophytic microbial assemblages. Such richness in recoverable isolates suggests that *A. octandra* var. *malesiana* constitutes a highly favorable ecological niche, fostering pronounced heterogeneity within its endophytic bacterial community [38]. The relatively high number of isolates recovered in this study is consistent with recent reports showing that tropical and ethnomedicinal plants tend to harbor rich microbial assemblages capable of synthesizing biologically active compounds [39, 40].

Endophytic communities in medicinal plants are increasingly recognized for their ecological and biochemical significance, as they often produce metabolites that contribute to host defense or environmental adaptation. Many species of endophytic bacteria are known to synthesize new secondary metabolites with significant activities, such as antibacterial, antifungal, antiseptic, antimicrobial, anticancer, and antiviral activities, highlighting their great bioactive potential [41, 42]. Antibacterial screening of 44 isolates from *A. octandra* var. *malesiana* leaf revealed that 12 strains were able to inhibit *S. pyogenes*, suggesting that only a subset possesses the metabolic pathways necessary to produce compounds with clinically relevant antibacterial properties. The variability in inhibition zone diameters further indicates metabolic heterogeneity among the isolates. Isolate AO4 exhibited the strongest inhibitory activity against *S. pyogenes*, producing the largest inhibition zone and highlighting its potential for further investigation as a source of antibacterial compounds.

The morphological and biochemical properties of potential Isolate AO4 demonstrated clear characteristics associated with the genus *Streptomyces*. The colony and microscopic features of AO4 are chalky texture, well‐developed aerial mycelia, filamentous hyphae, and rectiflexible spore chains—supporting its classification within the *Streptomyces* lineage. These traits correspond with established morphological descriptions of antibiotic‐producing actinomycetes [43]. Actinomycetes are recognized as prolific producers of diverse antibiotics, contributing significantly to major classes of antibacterial agents, including *β*‐lactams, aminoglycosides, tetracyclines, macrolides, glycopeptides, and ansamycins [44]. Comparable observations have been documented in actinomycetes, where certain strains exhibit disproportionately strong antimicrobial activity due to enhanced expression of secondary metabolite pathways [45–47]. The observed phenotypic traits of AO4 suggest metabolic versatility and the ability to produce compounds that may underlie its antibacterial activity.

The 16S rRNA gene is the most extensively employed molecular marker for bacterial systematics, particularly among actinomycetes, owing to its high phylogenetic resolution and comprehensive representation in curated sequence databases [48]. The molecular identification based on 16S rRNA sequencing placed Isolate AO4 within the genus *Streptomyces*. The strong sequence homology observed in this study supports the morphological and biochemical traits previously recorded for AO4, reinforcing the reliability of its identification as *S. luteolus*. Members of *S. luteolus* and its closely related phylogenetic lineages are well recognized for their capacity to biosynthesize a wide range of bioactive metabolites, including antimicrobial agents, siderophores, and signaling molecules [49, 50].

LC–MS/MS profiling revealed the production of two primary metabolites, 4‐DMAP and nicotinic acid. 4‐DMAP is more commonly discussed in the context of chemical catalysis; several recent studies have reported pyridine‐based microbial metabolites and derivative compounds with antibacterial activity [51, 52]. Nicotinic acid is also widely distributed across microbial taxa and has been associated with antibacterial, anti‐inflammatory, and immunomodulatory functions [53–55].

In silico docking provided insights into the potential mechanisms of action of AO4 metabolites against *S. pyogenes*. Bioactivity prediction showed that 4‐DMAP inhibits several activities to molecular metabolism of bacteria including NAD(P)+‐arginine ADP‐ribosyltransferase inhibitor (Pa = 0.592), alcohol dehydrogenase inhibitor (Pa = 0.213), pyruvate dehydrogenase inhibitor (Pa = 0.168), aldehyde dehydrogenase (NADP+) inhibitor (Pa = 0.303), NADH dehydrogenase inhibitor (Pa = 0.139), acyl‐CoA oxidase inhibitor (Pa = 0.234), aldehyde dehydrogenase inhibitor (Pa = 0.149), and malate‐CoA ligase inhibitor (Pa = 0.196). Moreover, nicotinic acid showed several inhibition activities to molecular metabolism of bacteria including NAD(P)+‐arginine ADP‐ribosyltransferase inhibitor (Pa = 0.74), alcohol dehydrogenase inhibitor (Pa = 0.351), pyruvate dehydrogenase inhibitor (Pa = 0.567), aldehyde dehydrogenase (NADP+) inhibitor (Pa = 0.765), NADH dehydrogenase inhibitor (Pa = 0.264), acyl‐CoA oxidase inhibitor (Pa = 0.327), aldehyde dehydrogenase inhibitor (Pa = 0.366), and malate‐CoA ligase inhibitor (Pa = 0.386).

Both 4‐DMAP and nicotinic acid were predicted to interact with essential bacterial targets, including DNA gyrase and PBP. Multitarget interaction patterns such as these have recently been reported as contributing to broader or more resilient activity in natural products [56]. 4‐DMAP and nicotinic acid demonstrated comparatively lower binding affinities than the control ligands in the molecular docking analysis. Although 4‐DMAP and nicotinic acid exhibited lower binding affinities than the control ligands, their interactions with key residues of the target proteins suggest that these metabolites may still contribute to the observed antibacterial activity. However, their specific roles remain speculative because the purified compounds were not individually evaluated against *S. pyogenes* and no bioassay‐guided fractionation was performed. The antibacterial activity of AO4 may arise from these metabolites, other unidentified compounds, or synergistic interactions among multiple constituents. Further studies involving purified compounds, MIC/MBC assays, and metabolite‐guided fractionation are required to clarify their individual contributions.

ADMET profiles justify considering them as promising hit‐like scaffolds worthy of further optimization. In modern drug discovery workflows, early ADMET evaluation encompassing absorption, distribution, metabolism, excretion, and toxicity has become an important dimension in drug discovery. ADMET evaluation is considered essential to reduce the high attrition rate of candidates with good in vitro potency but poor pharmacokinetic or safety profiles [57]. Poor pharmacokinetic or toxicity profiles are among the leading causes of attrition in preclinical and clinical stages [58, 59].

Both compounds show excellent predicted oral absorption, with high solubility and human intestinal absorption (HIA) suggesting efficient uptake via the gastrointestinal tract. High fraction unbound values further suggest that a substantial proportion of the administered dose remains free in plasma, thereby increasing the probability of reaching and interacting with the target enzyme. Importantly, 4‐DMAP and nicotinic acid are not predicted to inhibit or act as substrates for major CYP450 isoforms, reducing the risk of metabolic liabilities or drug–drug interactions, a frequent obstacle in lead development [60]. Both compounds show low predicted toxicity: no hepatotoxicity, negative in mutagenicity tests (e.g., AMES), and relatively high tolerated dose thresholds (e.g., LD_50_).

The control ligands (ciprofloxacin and ampicillin) showed hepatotoxicity. Ciprofloxacin has been reported to be heavily implicated in liver disease [61]. A comprehensive review highlights that a wide range of therapeutic agents, including antibiotics, nonsteroidal anti‐inflammatory drugs (NSAIDs), and antituberculosis medications, are frequently associated with hepatotoxic outcomes [62]. Clinical evidence shows that antibiotics and antituberculosis agents are among the most commonly implicated drugs in reported cases of hepatotoxicity worldwide [63]. The absence of hepatotoxicity signals in the in silico profiles of the candidate molecules highlights a noteworthy safety advantage, underscoring their suitability for further progression and optimization in drug discovery.

The metabolites identified in *S. luteolus* AO4 (4‐DMAP and nicotinic acid) feature pyridine‐based scaffolds, which are critical structural elements in modern pharmaceuticals due to their diverse biological roles [64]. Recent research highlights that pyridine‐based agents can selectively inhibit bacterial ATP synthase, an essential enzyme in cellular bioenergetics [65]. Furthermore, pyridine derivatives such as oxazolidinones demonstrate strong antibacterial efficacy by binding to the 50S ribosomal subunit at the peptidyl transferase center (PTC) [66].

The antimicrobial potential of these pyridine‐containing compounds is often enhanced by structural features like the azomethine group, which facilitates hydrogen bonding with the active sites of bacterial enzymes and increases lipophilicity for better membrane penetration [67]. The presence of these specific scaffolds in *S. luteolus* AO4 provides a promising foundation for developing novel therapeutic agents against Gram‐positive pathogens, including those associated with tonsillitis.

## 5. Conclusions

Isolation of endophytic bacteria from the native Sumatra plant, *A. octandra* var. *malesiana*, resulted in 44 isolates, with 12 strains exhibiting antibacterial activity against *S. pyogenes*. The most potent isolate, AO4, was characterized based on morphological and biochemical characteristics and identified as *S. luteolus* based on 16S rRNA analyses. Metabolomic profiling revealed two dominant metabolites—4‐DMAP and nicotinic acid—while in silico evaluation demonstrated their interactions with key bacterial targets, including DNA gyrase and PBP, with promising pharmacokinetic properties. These findings suggest that endophytic bacteria associated with *A. octandra* var. *malesiana*, particularly *S. luteolus* AO4, represent potential sources of antibacterial metabolites and warrant further investigation for their possible application against tonsillitis‐associated pathogens.

## Funding

This study was funded by the Kementerian Riset, Teknologi dan Pendidikan Tinggi, 10.13039/501100010447, 102/C3/DT.05.00/PL/2025.

## Conflicts of Interest

The authors declare no conflicts of interest.

## Supporting information


**Supporting Information** Additional supporting information can be found online in the Supporting Information section. File S1 This file contains detailed information on docking site information and molecular docking parameters, including docking grid centers, dimensions, and target binding site residues for DNA gyrase (PDB ID: 1KZN) and penicillin‐binding protein (PDB ID: 3HUN) used in this study.

## Data Availability

The data that supports the findings of this study are available in the supporting information of this article.
