# Supplementary material for: Potential Antitonsillitis Metabolites From Endophytic Bacteria Associated With Aporosa octandra var. malesiana: A Metabolomic and Molecular Docking Study
Source: Int J Microbiol. 2026 Jul 17;2026:7582229. doi: 10.1155/ijm/7582229 (PMC13377793; doi:10.1155/ijm/7582229)
Supplement: Supplementary file 1 — Supporting Information Additional supporting information can be found online in the Supporting Information section. File S1 This file contains detailed information on docking site information and molecular docking parameters, including docking grid centers, dimensions, and target binding site residues for DNA gyrase (PDB ID: 1KZN) and penicillin‐binding protein (PDB ID: 3HUN) used in this study. [file IJM-2026-7582229-s001.docx]

**Supplementary 1**. Docking sites of each protein

| **Proteins** | **Center** | | | **Dimensions (Amstrong)** | | | **Target Residues** |
| --- | --- | --- | --- | --- | --- | --- | --- |
|  | **x** | **y** | **z** | **x** | **y** | **z** |  |
| DNA gyrase (PDB ID: 1KZN) | 19,3551 | 19,463 | 38,2388 | 23,1589 | 37,7433 | 31,6758 | Arg22, Tyr26,  Glu42 Val43,  Asp45, Asn46,  Ala47, Asp49,  Glu50, Ile59,  Val71, Gln72,  Asp73, Gly75,  Arg76, Gly77,  Ile58, Pro79,  Ala86, Ile90,  Met91, Val93,  Leu94, His95,  Ala 96, Gly117,  Val118, Gly119,  Val120, Ser121,  Val122, Arg136,  Gly164, Thr165,  Met166, Val167 |
|  |  |  |  |  |  |  | (Lafitte et al, 2002 (https://doi.org/10.1021/bi0159837);  Abdul‑Hammed et al, 2024 (https://doi.org/10.1007/s40203-024-00234-z)) |
| Penicilin binding protein (PDB ID:3HUN) | -22,576 | 5,3014 | -3,8241 | 12,7797 | 18,9208 | 15,1408 | Ser75, Ser116,  Ser139, Phe241,  Thr260, Gly261,  Ser262, Glu297 |
|  |  |  |  |  |  |  | (Navara et al, 2010 (https://doi.org/10.1128/jb.00822-09)) |
